# Host Susceptibility Modulates *Escovopsis* Pathogenic Potential in the Fungiculture of Higher Attine Ants

**DOI:** 10.3389/fmicb.2021.673444

**Published:** 2021-06-14

**Authors:** Irina Jiménez-Gómez, Mariana O. Barcoto, Quimi V. Montoya, Aryel C. Goes, Lana S. V. E. Monteiro, Odair C. Bueno, Andre Rodrigues

**Affiliations:** ^1^Department of General and Applied Biology, São Paulo State University (UNESP), Rio Claro, Brazil; ^2^Centro de Investigación en Dinámica Celular, Universidad Autónoma del Estado de Morelos, Cuernavaca, Mexico

**Keywords:** host-pathogen interactions, opportunistic infections, pathogenesis, commensals, dysbiosis, host resistance, colony defenses

## Abstract

Health and disease emerge from intricate interactions between genotypes, phenotypes, and environmental features. The outcomes of such interactions are context-dependent, existing as a dynamic continuum ranging from benefits to damage. In host-microbial interactions, both the host and environmental conditions modulate the pathogenic potential of a microorganism. Microbial interactions are the core of the agricultural systems of ants in the subtribe Attina, which cultivate basidiomycete fungi for food. The fungiculture environment harbors a diverse microbial community, including fungi in the genus *Escovopsis* that has been studied as damage-causing agent. Here, we consider the ant colony as a host and investigate to what extent its health impacts the dynamics and outcomes of host-*Escovopsis* interactions. We found that different ant fungal cultivars vary in susceptibility to the same *Escovopsis* strains in plate-assays interactions. In subcolony-*Escovopsis* interactions, while healthy subcolonies gradually recover from infection with different concentrations of *Escovopsis* conidia, insecticide-treated subcolonies evidenced traits of infection and died within 7 days. The opportunistic nature of *Escovopsis* infections indicates that diseases in attine fungiculture are a consequence of host susceptibility, rather than the effect of a single microbial agent. By addressing the host susceptibility as a major modulator of *Escovopsis* pathogenesis, our findings expand the understanding of disease dynamics within attine colonies.

## Introduction

Dynamics in microbial interactions derive from trade-offs between the interacting organisms, modulated by diverse environmental variables (Gonze et al., 2018). Outcomes that emerge from interacting genotypes, phenotypes, and environmental conditions, exist as a dynamic continuum (Méthot and Alizon, 2014). This includes benefits, damage, or be neutral to the interacting organisms, ultimately resulting in ecological states of mutualism, commensalism, colonization, and disease (Frey-Klett et al., 2011; Casadevall and Pirofski, 2015). Such ecological categories have no fixed boundaries, in a way that the same organism could be considered a commensal and a pathogen, depending on the ecological circumstances and other interacting species (Méthot and Alizon, 2014; Casadevall, 2017). In host-pathogen interactions, host features as susceptibility, resistance, and tolerance to infection modulate the interaction outcomes, even determining the infectivity success (Thompson, 1986; Best et al., 2009). Susceptibility refers to environmental attributes within and outside the host that favor the establishment, development, and maturation of a pathogen (Wakelin, 1978; Méthot and Alizon, 2014; Casadevall and Pirofski, 2018). These variables include host genetics, health status, and microbiome, as well as climatic conditions and chance of being infected (Casadevall and Pirofski, 2018). By mechanisms of tolerance or resistance, the host is able to limit, prevent, or reduce the probability of infection at any stage of the interaction (Wakelin, 1978; Antonovics et al., 2013; Vale et al., 2014). While a tolerant host might employ mechanisms to improve its own health to tolerate the infection (without interfering directly with the pathogen), a resistant host might directly target the pathogen and its derived toxins (Vale et al., 2014).

Cumulative evidence indicates that both the host and the ecological conditions modulate the pathogenic potential of a microorganism (Vale et al., 2014; Casadevall, 2017). Thus, the interaction dynamics determine the expression of infectivity attributes, which comprise features allowing the infection and multiplication within a host (Antonovics et al., 2013; Méthot and Alizon, 2014). Host-pathogen relationships have been historically studied through a pathogen-centric view (Casadevall and Pirofski, 1999, 2003). However, centering the pathogen as the sole disease-driving agent narrows the comprehension of complex ecological and evolutionary processes (Little et al., 2010; Méthot and Alizon, 2014). Health and disease are end results of intertwined processes within a network of players that include the host, pathogen, other members of the microbiota, and environmental properties (Restif and Koella, 2003; Lambrechts et al., 2006; Méthot and Alizon, 2014; Casadevall and Pirofski, 2015). Host-microbial interactions achieve an additional level of complexity in animal societies, where dense aggregations of individuals may favor the dispersion of both beneficial and harmful microorganisms (Biedermann and Rohlfs, 2017; Bratburd et al., 2020). Strategies to maintain and propagate beneficial microbes, as well as to avoid and inhibit the harmful ones, are thought as an important aspect of insects’ social evolution (Biedermann and Rohlfs, 2017).

Beneficial and harmful host-microbial interactions are at the core of the agricultural systems of fungus-growing ants in the subtribe Attina (Hymenoptera: Formicidae: Attina; Mueller et al., 2005; Biedermann and Rohlfs, 2017). These ants evolved an obligate mutualistic association with basidiomycete fungi cultivated in ant colonies for food (Mueller et al., 2005). Ant workers forage for leaves, seeds, insect frass and carcasses to nourish their symbiotic fungus in the fungus gardens (Martin, 1970; De Fine Licht and Boomsma, 2010). Fungal hyphae grow within the fungus garden by metabolizing the substrate, providing all nourishment for ant larvae, queen, and part of the nourishment for adult ant workers (Quinlan and Cherrett, 1979; Silva et al., 2003; Moller et al., 2011; De Fine Licht et al., 2014). The fungus garden also harbors a wide diversity of microorganisms (Fisher et al., 1996; Carreiro et al., 1997; Rodrigues et al., 2008; Aylward et al., 2012; Barcoto et al., 2020), including filamentous fungi in the genus *Escovopsis* (Ascomycota: Hypocreales; Currie et al., 1999a). *Escovopsis* was described as a specialized parasite to the ant-fungus symbiosis (Currie et al., 2003), associated with decrease in fungus garden biomass and number of workers and brood (Currie, 2001).

Patterns of phylogenetic congruence suggested a tripartite coevolution between the ants, the cultivated fungi, and *Escovopsis* (Currie et al., 2003; Gerardo et al., 2006a). Mechanisms of interaction (Reynolds and Currie, 2004; Folgarait et al., 2011; Marfetán et al., 2015; Varanda-Haifig et al., 2017), patterns of virulence (Currie, 2001; Wallace et al., 2014; Marfetán et al., 2015), patterns of specificity (Gerardo et al., 2004, 2006b; Taerum et al., 2007; Birnbaum and Gerardo, 2016), and detrimental impact of infection in ant colonies (Currie, 2001) have been evaluated mainly through an *Escovopsis*-centered perspective. Here, considering the ant colony as a host (composed by the fungus garden and the ants), we evaluate host-*Escovopsis* interactions in the fungiculture of higher attines. Our first approach assessed *in vitro* interactions between *Escovopsis* and the symbiotic fungi cultivated by the higher attines *Atta sexdens* and *Mycetomoellerius tucumanus*. In the second approach, we analyzed interactions between *Escovopsis* and queen-less colonies of *A. sexdens* containing ant workers, pupae, and larvae (hereafter mentioned as subcolonies). Specifically, we investigate (i) whether fungal cultivars are differentially susceptible to diverse *Escovopsis* strains; (ii) whether fungal cultivar-*Escovopsis* interactions (FEI) follow a phylogenetic distribution; (iii) how healthy subcolonies respond to infection with *Escovopsis* conidia in different concentrations; and (iv) how insecticide-treated subcolonies respond to *Escovopsis* conidia infection. Our findings evidenced the cultivar and the subcolony susceptibility as important modulators of the dynamics and outcomes of subcolony-*Escovopsis* interactions (SEI) in the higher attine symbiosis. When the ants and fungus-gardens are healthy, effective defenses are built preventing *Escovopsis* infections.

## Materials and Methods

### Fungal Strains

Fungal cultivar-*Escovopsis* interactions were examined using 21 *Escovopsis* strains, distributed across the phylogeny. These strains were isolated from fungus gardens of higher attine ants in the genera *Atta*, *Acromyrmex*, *Mycetomoellerius*, *Paratrachymyrmex*, and *Trachymyrmex sensu lato* (Table 1), and were phylogenetically analyzed by Meirelles et al. (2015). Strains were revived prior to the experiments by inoculating cryopreserved conidia on potato dextrose agar (PDA, Neogen Culture Media, Lansing, MI, United States) and incubating at 25°C for 7 days in the dark. Such conditions are reported to favor *Escovopsis* development (Montoya et al., 2019). The experiments included two strains of fungal cultivars: *Leucoagaricus gongylophorus* FF2006, obtained in 2006 from fungus gardens of an *A. sexdens* colony (*L. gongylophorus* AS) maintained at the Center for the Study of Social Insects (CEIS, UNESP); and *Leucoagaricus* sp. IJ2016, obtained in 2016 from fungus gardens of a *M. tucumanus* (*Leucoagaricus* sp. MT) colony collected at the UNESP campus. Strain IJ2016 was isolated on PDA supplemented with 150 μg mL^–1^ of chloramphenicol (Sigma, St. Louis, MO, United States). Both fungal strains are maintained in the laboratory under continuous transfer on culture medium (as described by Pagnocca et al., 1990). Cultures are considered vigorous since they fully develop staphyla and gongylidia on artificial medium.

**TABLE 1:** *Escovopsis* strains isolated from fungus gardens of higher attine ants in Brazil and other fungal species used in the phylogenetic analyses.

**LESF ID^1^**	**ID^1^**	**Fungi**	**Ant species^2^**	**Ant colony ID**	**City/State^3^**	**GenBank accessions^4^**	
LESF 018	NL002	*Escovopsis* sp.	*Atta capiguara*	N66	Botucatu/SP	KM817143	KM817073
LESF 021	ES002	*Escovopsis* sp.	*Atta sexdens*	N-Ale	Rio Claro/SP	KM817123	KM817053
LESF 022	ES003	*Escovopsis* sp.	*Atta cephalotes*	SES040121-01	Frei Caneca/PE	KM817124	KM817054
LESF 037	ES033	*Escovopsis* sp.	*Atta cephalotes*	CTL110912-05	Parauapebas/PA	KM817141	KM817071
LESF 044	RS061	*Escovopsis* sp.	*Acromyrmex heyeri*	AOMB110904-15	Pelotas/RS	EU082799	KM817081
LESF 045	RS076	*Escovopsis* sp.	*Acromyrmex coronatus*	AOMB130904-04	Vacaria/RS	EU082801	KM817082
LESF 046	SES001	*Escovopsis* sp.	*Trachymyrmex* sp. *sensu lato*	SES080402-03	Rio Claro/SP	KM817146	KM817084
LESF 049	SES007	*Escovopsis* sp.	*Mycetomoellerius kempfi*	SES080921-03	Uberlândia/MG	KM817151	KM817090
LESF 050	SES008	*Escovopsis* sp.	*Acromyrmex* sp.	SES081007-01	São Sebastião/RO	KM817152	KM817091
LESF 051	SES009	*Escovopsis* sp.	*Mycetomoellerius fuscus*	SES081108-04	Palmeiras/BA	KM817153	KM817092
LESF 052	SES010	*Escovopsis* sp.	*Paratrachymyrmex diversus*	SES090109-06	Manaus/AM	KM817154	KM817093
LESF 106	SES006	*Escovopsis* sp.	*Mycetomoellerius dichrous*	SES080922-02	Uberlândia/MG	KM817150	KM817089
LESF 135	SES003	*Escovopsis* sp.	*Trachymyrmex* sp. *sensu lato*	CTL080820-02	Uberlândia/MG	KM817148	KM817086
LESF 315	NL007	*Escovopsis* sp.	*Atta sexdens*	N68	Botucatu/SP	KF240730	KM817075
LESF 316	ES001	*Escovopsis* sp.	*Trachymyrmex* sp. *sensu lato*	TR-117	Rio Claro/SP	KM817122	KM817052
LESF 317	ES026	*Escovopsis* sp.	*Mycetomoellerius tucumanus*	ARTD030908-02	Rio Claro/SP	KM817137	KM817067
LESF 318	ES029	*Escovopsis* sp.	*Acromyrmex* sp.^5^	WGPM091021-01	Palmas/TO	KM817139	KM817069
LESF 319	ES030	*Escovopsis* sp.	*Acromyrmex* sp.^5^	AR091020-01	Palmas/TO	KM817140	KM817070
LESF 325	BA004	*Escovopsis* sp.	*Atta cephalotes*	BMSR120703-01(FL5)	Camacan/BA	KM817119	KM817119
LESF 326	BA006	*Escovopsis* sp.	*Atta cephalotes*	BMSR120803-01(CA10)	Camacan/BA	KM817049	KM817049
LESF 858	BA001	*Escovopsis* sp.	*Atta cephalotes*	BMSR120702-01(FL1)	Camacan/BA	KM817116	KM817046
	CBS 35085	*Lecanicillium antillanum*				DQ522350	NR111097
	CTR77155	*Trichoderma avellaneum*				AY225857	DQ020000
	P1	*Trichoderma atroviride*				EF581849	AF278794
	TFC200723	*Hypomyces samuelsii*				FN868769	FN859451
	CLL7259	*Hypomyces samuelsii*				FN868764	FN859445
	CBS70588	*Hypomyces semicirculare*				FN868735	NR121425
	CBS67677	*Hypomyces asterophorum*				FN868712	NR111426
	TFC201316	*Hypomyces protrusum*				FN868732	FN859414

*^1^IDs: *Escovopsis* strains kept at the Laboratory of Fungal Ecology and Systematics – LESF (UNESP, Rio Claro, SP) and corresponding strain IDs used in Meirelles et al. (2015). Strain IDs for the other fungi were obtained from GenBank. ^2^*Trachymyrmex* classification follows Solomon et al. (2019). See Supplementary Material of that paper for colony IDs that overlap with the ones of this study. ^3^AM: Amazonas; BA: Bahia; MG: Minas Gerais; PA: Pará; PE: Pernambuco; SP: São Paulo; RO: Rondônia; RS: Rio Grande do Sul; TO: Tocantins. ^4^Sequences of *Escovopsis* strains were generated in Meirelles et al. (2015) and are deposited in GenBank. ^5^These two collections were reidentified as *Acromyrmex* sp. but were mistakenly labeled as *Trachymyrmex* sp. in Meirelles et al. (2015).*

### Dual-Culture Assays

Fungal cultivar-*Escovopsis* interactions were analyzed through dual-culture assays (Silva et al., 2006; Varanda-Haifig et al., 2017). Briefly, the fungal cultivar (either *L. gongylophorus* AS or *Leucoagaricus* sp. MT) was grown for 18 days on PDA, at 25°C in the dark. From these cultures, a mycelium fragment of 0.8 cm in diameter was cut and inoculated on PDA, at 15 mm from the border of the Petri dish (Supplementary Figure 1). These plates were maintained at 25°C for 15 days in the dark. After the head-start growth of the fungal cultivar, one mycelium fragment of *Escovopsis* was inoculated 30 mm from the border of the ant fungal cultivar colony. *Escovopsis* inoculum was obtained from a culture previously grown on PDA (at 25°C for 7 days in the dark). For this experiment, two control sets of were included: (i) plates inoculated only with the fungal cultivar (either *L. gongylophorus* AS or *Leucoagaricus* sp. MT) and (ii) plates inoculated only with *Escovopsis*. Each tested strain and control had eight technical replicates, which were incubated at 25°C for 14 days in the dark. We used the colony growth area (in mm^2^) to infer the effect of *Escovopsis* on the fungal cultivar growth and for evaluating the *Escovopsis* growth rate. The growth area of the fungal cultivar was measured immediately before *Escovopsis* inoculation and after 1, 2, 3, 5, 7, 10, and 14 days of incubation. Colony measurements were carried out from images of digitized plates (HP Deskjet 2050-J510 scanner) in ImageJ 1.x (Schneider et al., 2012).

#### Fungal Cultivar and *Escovopsis* Growth Inhibition

Fungal cultivar-*Escovopsis* interactions outcomes were based on the mean values of the final area of colony growth for both fungi. Measurements were taken for all interaction plates (FEI) and for all control plates (fungal cultivar and *Escovopsis* growing separately). Mean values of growth areas were reported with 95% confidence intervals using a null model developed by a Monte Carlo method with 10,000 randomizations. This analysis was performed in PopTool v3.2 (*p* < 0.05).

For each day of experiment, growth areas in the interaction plates were standardized by the mean values reported for control plates. The mean growth area of the fungal cultivar in the presence of *Escovopsis* were standardized by the mean growth area values of the fungal cultivar in the control plates (Supplementary Tables 1, 2). The mean growth area of *Escovopsis* in the presence of fungal cultivars were standardized by the mean values of growth areas of *Escovopsis* in the control plates (Supplementary Tables 3, 4). Then, the standardized growth areas in the interaction plates were compared with the expected value of the control plates (area equals to 1 in a t distribution) using a *t*-test for single means (*p* < 0.05) in Statistica v8.0.360.

#### Fungal Cultivar-*Escovopsis* Interaction Patterns

Fungal cultivar-*Escovopsis* interactions were classified according to the mycelial growth inhibition of the fungus after 14 days of experiment. Standardized values (Supplementary Tables 1, 2) were used to estimate the fungal cultivar mycelial growth inhibition (%) by

$${\text{Cultivar}}_{\text{control}}-{\text{Cultivar}}_{\text{interacting}}*100$$

Where:

Cultivar_control_: Fungal cultivar mycelia area in control plates (standardized final growth area)

Cultivar_interacting_: Fungal cultivar mycelia area in interaction plates (standardized final growth area).

**FIGURE 1:**
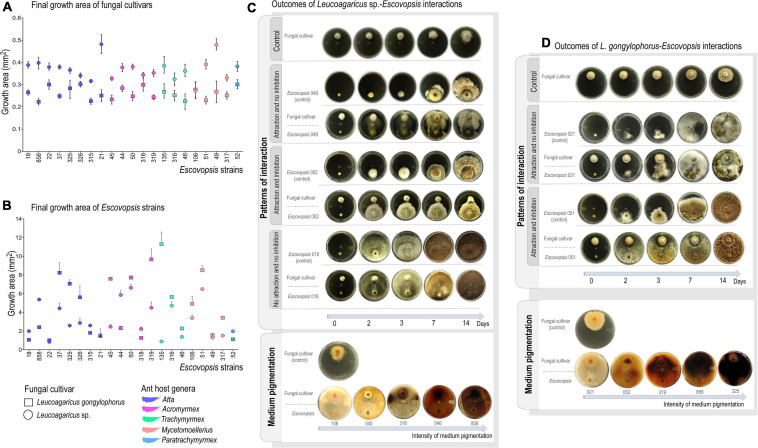
Interaction outcomes on plate-assays. **(A)** Final growth area (in mm^2^) of *Leucoagaricus gongylophorus* and *Leucoagaricus* sp. after interacting with *Escovopsis* strains for 14 days. **(B)** Final growth area of *Escovopsis* strains after interacting with fungal cultivars for 14 days. Bars correspond to 95% of confidential interval under Monte Carlo regression analysis (1,000 randomizations). **(C)** Classification of interactions outcomes between *Leucoagaricus* sp. and *Escovopsis*, which include the patterns of interaction observed and pigmentation of culture media. **(D)** Classification of interactions outcomes between *L. gongylophorus* and *Escovopsis*, including patterns of interaction and pigmentation of culture media.

Obtained values fell between 0% (no inhibition of fungal cultivar growth) and 100% (total inhibition of fungal cultivar growth). Growth inhibition of *L. gongylophorus* AS or *Leucoagaricus* sp. MT was compared by Mann-Whitney non-parametric test (*p* < 0.05) in PAST 3 (Hammer et al., 2001). Inhibition patterns were classified according to Birnbaum and Gerardo (2016) as: (i) attraction and no inhibition: *Escovopsis* grows directly toward the fungal cultivar, and it is not inhibited; (ii) attraction with inhibition: *Escovopsis* grows directly toward the fungal cultivar, but its own growth is inhibited; (iii) no attraction and no inhibition: *Escovopsis* neither grows directionally toward the fungal cultivar nor has its growth inhibited (Figure 1).

### Correlation Between Outcomes of Fungal Cultivar-*Escovopsis* Interactions and *Escovopsis* Phylogeny

To evaluate whether patterns of FEI have any phylogenetic underpinnings, interaction outcomes were pinpointed in the *Escovopsis* phylogeny. Phylogenetic analysis was performed combining 52 sequences of the internal transcriber spacer region (ITS) and of the elongation factor 1 alpha (*tef*1) gene. *Escovopsis* sequences were obtained from Meirelles et al. (2015). Sequences of two *Cladobotryum*, three *Hypomyces* and two *Trichoderma* strains were included as close relatives of *Escovopsis* and *Lecanicillium antillanum* was used as the outgroup (Montoya et al., 2019). GenBank accessions of the 21 strains used in the assays, seven strains used as closest relatives to *Escovopsis* and one strain used as outgroup are listed in Table 1. The ITS and *tef*1 data sets were aligned independently using MAFFT v. 7 (Katoh and Standley, 2013), edited manually, and subsequently concatenated using WINCLADA v. 1.00.08 (Nixon, 1999). The final data set contained 1,384 bp (ITS – 626 bp, and *tef*1 – 758 bp). Bayesian inference was applied to reconstruct the final phylogenetic tree in MrBayes v.3.2.2 (Ronquist et al., 2012), applying GTR as nucleotide substitution model for both markers independently under 95% of confidence interval of Bayesian information criterion, in jModelTest 2 (Darriba et al., 2012). Two independent runs were carried out, using three hot chains and one cold chain. A Markov Chain Monte Carlo (MCMC) sampling of two million generations was sufficient for achieving standard deviation values of split frequencies below 0.01. The first 25% of MCMC generations were discarded as “burn-in.” The final phylogenetic tree was edited in FigTree v.1.4 and Adobe Illustrator CC v.17.1.

### Infection Assays

Subcolonies of *A. sexdens* ants, which cultivate *L. gongylophorus* (Weber, 1966; Mueller et al., 2017), were infected with *Escovopsis* conidia for assessing SEI. All subcolonies used in the infection assays were set up using workers and fungus garden sampled from healthy *A. sexdens* colonies (1–2 years old). The experimental system consisted of three chambers (500 mL disposable plastic containers) connected by plastic tubes: one for the foraging chamber, the central one for the fungus garden chamber, and one for the waste disposal (Supplementary Figure 2). A thin layer of Teflon^®^ (polytetrafluoroethylene) was applied to the inner walls of the containers to prevent workers from escaping, and a layer of moistened plaster was added to the garden chamber for humidity preservation. A fragment of fungus garden (19–22 g) was carefully placed in the central chamber. Due to difficulties in determining the exact number of workers without disrupting the fungus garden structure (Currie, 2001), their number was not precisely specified. Though, fungus garden contained all worker castes (including minor, media, and major workers), eggs, larvae, and pupae in different stages of development. Subcolonies were acclimatized for 14 days before the experiment at 23–25°C, receiving *Hibiscus* sp. leaves *ad libitum* every 48 h. After acclimatized, subcolonies were randomly assigned to different treatments.

#### Infection of Healthy Subcolonies With *Escovopsis* Conidia

For the infection assay, 30 healthy colonies maintained in the laboratory were sampled to set up 90 subcolonies (Supplementary Figure 2). *Escovopsis* strains LESF 021, LESF 046, LESF 315, and LESF 318 were selected for this assay based on: (i) ant species from which *Escovopsis* strains were isolated (*A. sexdens*: LESF 021 and LESF 315; *Acromyrmex* sp. LESF 318, and *Trachymyrmex* sp. LESF 046; (ii) *Escovopsis* phylogenetic disposition of the strains: clade I (LESF 315 and LESF 046), clade II (LESF 318), and clade V (LESF 021), according to Meirelles et al. (2015); (iii) fungal cultivar growth inhibition observed in dual-culture assays; and (iv) interaction patterns observed in dual-culture assays: LESF 021 and LESF 318 (attraction and no inhibition); LESF 315 (attraction with inhibition); LESF 046 (no attraction and no inhibition).

Conidia suspensions of each *Escovopsis* strain were applied in four different concentrations, prepared from *Escovopsis* colonies grown for 7 days on PDA at 25°C in the dark. Test and control groups consisted of five randomly assigned subcolonies. Mycelial mass and conidia were suspended in 10 mL of 0.05% Tween 80. Conidia were separated from hyphal fragments in the suspension by filtering two to three times as previously described (Newmeyer, 1990; Osti and Rodrigues, 2018). The suspension was diluted to (i) 2–3 × 10^3^ conidia mL^–1^, (ii) 4.3–4.6 × 10^4^ conidia mL^–1^, (iii) 3.0–3.2 × 10^5^ conidia mL^–1^, and (iv) 2.1–2.2 × 10^6^ conidia mL^–1^, verified in a Neubauer chamber. Treatment was inoculated directly on the fungus garden surface using a hand sprayer: while test groups received 1 mL of conidia suspension, the control group received 1 mL of 0.05% Tween 80. Subcolonies were maintained in the same conditions described for acclimation for 14 days. Subcolony health conditions and foraging activity were scored daily by adapting a predefined score scale (Barcoto et al., 2017), and were statistically evaluated on each day separately using Friedman’s two-way ANOVA by ranks test in R v.3.1.0.

#### Infection of Insecticide-Treated Subcolonies With *Escovopsis* Conidia

The influence of subcolony susceptibility in SEI was evaluated by comparing the response of both healthy and insecticide-treated subcolonies to *Escovopsis* infection. Insecticide treatment was intended to disrupt subcolonies defenses by causing death of ant workers, through the application of 0.5 g of sulfluramid (Mirex-S^®^, insecticide in commercial bait) after 13 days of acclimation. For the artificial infection, LESF 046 (*Trachymyrmex* sp.) and LESF 318 (*Acromyrmex* sp.) were selected for having a slightly higher impact on healthy subcolonies in the previous assay. Conidia suspension of *Escovopsis* strains were applied subsequent to insecticide treatment.

The experimental setup included 48 subcolonies (assembled from 31 healthy lab colonies) assigned to six groups (each containing eight subcolonies). (i) Control: healthy subcolonies that received 1 mL of 0.05% Tween 80 solution; (ii) *Escovopsis* sp. LESF 318 (*Acromyrmex* sp.): healthy subcolonies that received 1 mL of LESF 318 conidia suspension – 7.1 × 10^6^ conidia mL^–1^; (iii) *Escovopsis* sp. LESF 046 (*Trachymyrmex* sp.): healthy subcolonies that received 1 mL of LESF 046 conidia suspension – 7.3 × 10^6^ conidia mL^–1^; (iv) Sulfluramid control: subcolonies treated with 0.5 g of sulfluramid that received 1 mL of 0.05% Tween 80 solution; (v) Sulfluramid + LESF 318 (*Acromyrmex* sp.): subcolonies treated with 0.5 g of sulfluramid that received 1 mL of LESF 318 conidia suspension – 7.1 × 10^6^ conidia mL^–1^; (vi) Sulfluramid + LESF 046 (*Trachymyrmex* sp.): subcolonies treated with 0.5 g of sulfluramid that received 1 mL of LESF046 conidia suspension – 7.3 × 10^6^ conidia mL^–1^). Conidia suspensions were prepared as described above. Subcolonies were maintained for 14 days in the same conditions set for acclimation period and scored daily (Barcoto et al., 2017). Fungi eventually overgrowing the fungus-garden were identified by morphological analysis (Montoya et al., 2019). Differences between groups were statistically evaluated on each day, using Friedman’s two-way ANOVA by ranks test in R v.3.1.0.

## Results

### Fungal Cultivars Are Differentially Susceptible to *Escovopsis*

**FIGURE 2:**
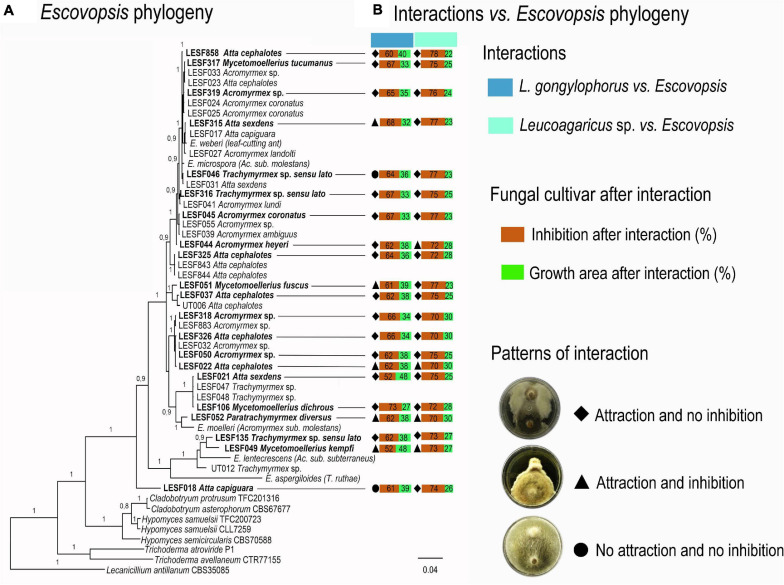
Phylogenetic distribution of fungal cultivar-*Escovopsis* interactions. **(A)** *Escovopsis* phylogeny; in bold *Escovopsis* strains used in the dual-culture assays. **(B)** Patterns of *L. gongylophorus*-*Escovopsis* and *Leucoagaricus* sp.-*Escovopsis* interactions. Growth (%) and inhibition (%) was calculated by comparing the final growth of the fungal cultivar when interacting with *Escovopsis* to the final growth area of the fungal cultivar growing solely. All final growth areas were standardized by the control final growth area mean values.

Both fungal cultivars had their mycelial growth inhibited in the presence of all the 21 *Escovopsis* strains in comparison to the control (*t* test, *p* < 0.05; Figure 1A; Supplementary Tables 1, 2). FEI resulted in 51.8 to 77.7% of cultivar mycelial growth inhibition (Supplementary Tables 1, 2), resulting in death in 92.8% of interactions. *Leucoagaricus* sp. MT tends to be more susceptible to inhibition by *Escovopsis* (73.9% of mycelial growth inhibition, Supplementary Table 1) than *L. gongylophorus* AS (63.1% of mycelial growth inhibition; Supplementary Table 2 and Figure 1A). Such pattern is observed for both interactions between same-ant-originating cultivar-*Escovopsis* (for instance: *L. gongylophorus* AS vs. LESF 315; *L. gongylophorus* AS vs. LESF 021; *Leucoagaricus* sp. MT vs. LESF 317), and for different-ant-originating cultivar-*Escovopsis* (Figure 1A). Differences in cultivar susceptibility are also supported by the final growth areas of *Leucoagaricus* sp. MT, which were significantly lower when compared to the final growth areas of *L. gongylophorus* AS (Mann-Whitney, *p* < 0.05; Figure 1A). Differences in susceptibility are also suggested by the day when inhibitions were first detected. *Leucoagaricus* sp. MT was significantly inhibited by seven *Escovopsis* strains after the first day in dual-culture (Supplementary Table 1), while *L. gongylophorus* AS inhibition was observed after 2 days (Supplementary Table 2). Some FEI were characterized by accumulation of soluble pigments (Figures 1C,D). Although the three patterns of interaction described by Birnbaum and Gerardo (2016) were observed (Figures 1C,D), the predominant was attraction and no inhibition (74% of fungal symbiont-*Escovopsis* interactions, Figure 2).

### *Escovopsis* Strains Grow Faster in the Presence of Fungal Cultivars

Interaction patterns do not seem clearly delimited across *Escovopsis* phylogeny (Figure 2A). *Leucoagaricus* sp. MT was most susceptible and tended to be more inhibited by *Escovopsis* regardless the *Escovopsis* strain origin, phylogenetic distribution, and interaction patterns (Figures 1, 2). A faster growth was observed for all *Escovopsis* strains when interacting with both fungal cultivars (*t*-test, *p* < 0.05; Figure 1B, Supplementary Tables 3, 4). *Escovopsis* strains tended to grow faster when interacting with *Leucoagaricus* sp. MT (171%, Supplementary Table 3) than in interactions with *L. gongylophorus* AS (162%, Figure 1B, Supplementary Table 4). However, *Escovopsis* final growth areas were not directly associated with percentage of fungal cultivar inhibition (Figure 2B). For instance, *Escovopsis* sp. LESF 044 (*Ac. heyeri*) and LESF 858 (*A. cephalotes*) presented high final growth areas (Figure 2B), though caused low fungal cultivar inhibition (Figures 2A,B).

### Healthy Fungus-Gardens Are Resistant to Infection With *Escovopsis* Conidia

**FIGURE 3:**
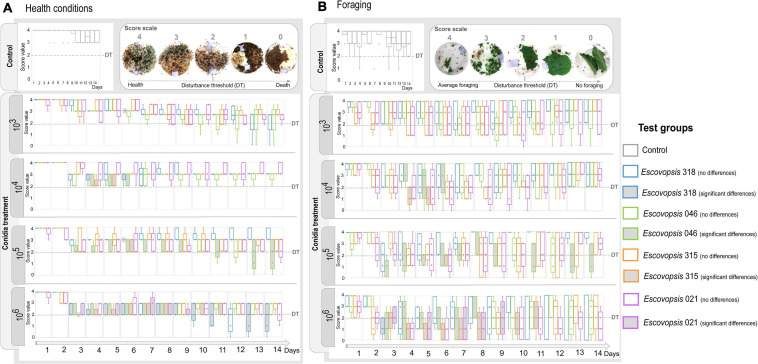
Changes in subcolonies followed through a score scale. **(A)** Health conditions and **(B)** foraging activity of *Atta sexdens* subcolonies when inoculated with different *Escovopsis* strains (LESF 046, LESF 318, LESF 315, and LESF 021) in different conidia concentrations (10^3^, 10^4^, 10^5^, and 10^6^ conidia mL^–1^), throughout 14 days of experiment. Boxes and markers in gray indicate significant differences when compared to the control in that specific day (Friedman’s two-way ANOVA by ranks, *p* < 0.05). Comparisons between test groups (Friedman’s two-way ANOVA by ranks *post hoc* test, *p* < 0.05) are available at Supplementary Table 6. Clear symptoms of disturbance were usually observed when subcolonies scored 2 in the score scale. This value was delimited as the Disturbance Threshold (DT), corresponding to the threshold amount of disturbance to become symptomatic.

While none of the subcolonies that received the control treatment died over 14 days of experiment, only four out of 80 (5%) subcolonies died when infected with different concentrations of *Escovopsis* conidia (Figure 3 and Supplementary Table 5). Of these four colonies, two were sprayed with conidia of LESF 046 (*Trachymyrmex* sp.) and two with LESF 318 (*Acromyrmex* sp.). None of the subcolonies treated with conidia of *Escovopsis* sp. LESF 021 (*A. sexdens*) and 315 (*A. sexdens*) died after 14 days. It is curious that even at high conidia concentration, most of the subcolonies were able to recover after 11 days of receiving the conidia treatment (Figure 3A). All *Escovopsis* strains significantly affected foraging activities in at least 1 day of experiment when 10^5^ and 10^6^ conidia mL^–1^ were inoculated (Figure 3B). Otherwise, none of the *Escovopsis* strains significantly impacted foraging activities when suspensions of 10^3^ conidia mL^–1^ were applied. Overall, subcolonies treated with all conidia concentrations gradually recovered before the experiment ending (Figure 3A).

### Insecticide-Treated Fungus-Gardens Are Susceptible to Infection With *Escovopsis* Conidia

**FIGURE 4:**
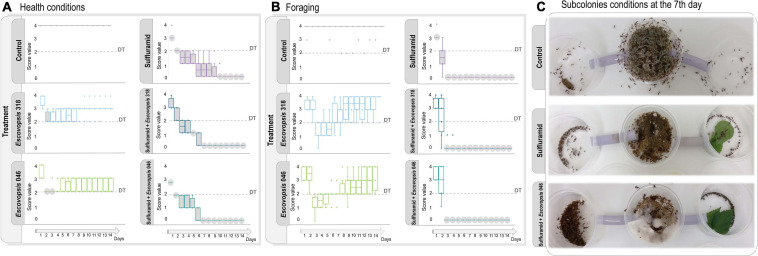
*Escovopsis* conidia effects in healthy and insecticide-treated *Atta sexdens* subcolonies. **(A)** Health conditions and **(B)** foraging activities after inoculation with *Escovopsis* strains LESF 046 and LESF 318, scored as defined in Figure 3. Boxes and markers in gray indicate significant differences when compared to the control in that specific day (Friedman’s two-way ANOVA by ranks, *p* < 0.05). Comparisons between test groups (Friedman’s two-way ANOVA by ranks *post hoc* test, *p* < 0.05) are available at Supplementary Table 8. Disturbance Threshold (DT) corresponds to the threshold amount of disturbance for the subcolonies to exhibit disease symptoms. **(C)** Subcolonies conditions at the 7th day after infection, when most of insecticide-treated subcolonies inoculated with *Escovopsis* conidia scoring as dead.

**FIGURE 5:**
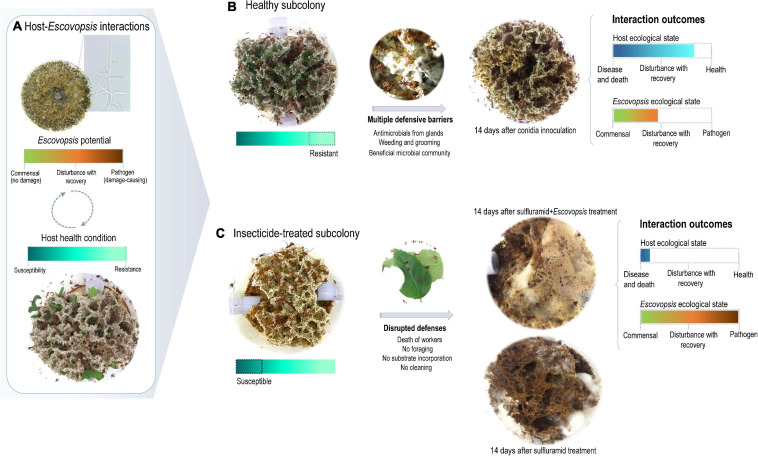
*Escovopsis* context-dependent pathogenesis. **(A)** Host-*Escovopsis* interactions would be a complex outcome deriving from interacting genotypes, phenotypes, and environmental features, resulting in a dynamic continuum ranging from benefits to damage, which would ultimately be modulated by the host susceptibility. **(B)** A healthy garden includes multiple defenses limiting the infectivity potential of *Escovopsis*. After 14 days of interaction, the garden shows no signals of infection or disease, being visibly similar to a healthy garden. In such interactions, *Escovopsis* could be acting as a commensal causing imperceptible damage to the host. **(C)** Insecticide-treated gardens present several disrupted defenses and the *Escovopsis* infection is not limited. In this context, *Escovopsis* would act as a pathogen damaging fungus garden health conditions fast and progressively.

Sulfluramid treatment increased ant mortality from the first day, and after 7 days the majority of ant workers were dying or dead (Figure 4 and Supplementary Table 8). All insecticide-treated subcolonies died by the end of the experiment (Figures 4A,B), meaning that foraging activity was not detected, the fungus garden was decayed, and most ants were dead or dying (Barcoto et al., 2017). For some subcolonies, the fungus garden was scored as decayed at the 7th day, even being overgrowth by *Escovopsis*, though presenting some survival ants (Figure 4C and Supplementary Table 8). Insecticide-treated subcolonies that were not inoculated with *Escovopsis* conidia had a significant decrease in healthy conditions and foraging activities already on the first day, dying on day 11 (Figures 4A,B). A similar pattern was observed in insecticide-treated subcolonies inoculated with *Escovopsis* conidia, which also experienced decline in health conditions on the first day of experiment, scoring as dead on day 7 (Figure 4A). Foraging activities were significantly reduced from the second day and completely interrupted from the third day (Figure 4B). Fungus gardens of these subcolonies were overgrown by the inoculated *Escovopsis* strains after the subcolony death (Figure 5), presenting also mycelia from non-inoculated *Escovopsis* strains, *Escovopsioides*, *Syncephalastrum, Fusarium*, and *Trichoderma* (Supplementary Table 6). Even though these subcolonies had died 4 days before the ones treated only with sulfluramid, there were no statistical differences in daily comparisons between them (Friedman’s two-way ANOVA by ranks *post hoc* test, *p* > 0.05; Supplementary Table 7).

In contrast, none of the healthy subcolonies inoculated with *Escovopsis* conidia died throughout 14 days of experiment. Although these subcolonies experienced a decline in health conditions after the second day, they gradually recovered and stabilized (Figure 4A). Similarly, foraging activities decreased after the second day of receiving *Escovopsis* conidia, and were gradually recovered about the seventh (*Escovopsis* sp. LESF 318 – *Acromyrmex* sp.) and eighth (*Escovopsis* sp. LESF 046 – *Trachymyrmex* sp.) day (Figure 4B).

## Discussion

The agricultural system of fungus-growing ants sustains diverse and complex microbial interactions, which determine the ecological success of this insect-microbial symbiosis (Currie et al., 1999a; Mueller et al., 2005; Fernández-Marín et al., 2006; Gerardo et al., 2006b; Poulsen et al., 2010; Suen et al., 2010; Aylward et al., 2012; Boya et al., 2017). As part of a dynamic continuum, whether these interactions would be beneficial, neutral, or harmful depend on the host health and the ecological conditions (Méthot and Alizon, 2014; Figure 5). *Escovopsis* fungi are reported as parasites of attine ants symbiosis (Currie et al., 1999a; Currie, 2001), and here we explore to what extent the susceptibility of the ants’ fungal cultivars and fungus gardens influence host-*Escovopsis* interactions. For being more susceptible than *L. gongylophorus* AS, *Leucoagaricus* sp. MT tend to be more inhibited by *Escovopsis.* These outcomes are not related to *Escovopsis* strains’ origin and interaction patterns, further evidencing that the cultivar susceptibility substantially impacts the interaction results (Figures 1, 2). The susceptibility of the colony (comprising the fungus gardens and ant workers) also seems fundamental in driving interactions outcomes, even determining the recovery and survival of subcolonies. While healthy subcolonies gradually recover from infection with different concentrations of *Escovopsis* conidia (Figure 3), insecticide-treated subcolonies become susceptible to infection and die within 7 days (Figure 4). However, it is unclear to what extent *Escovopsis* contributed to the death of subcolonies, which were already damaged by the insecticide effect. Thus, host susceptibility seems to modulate microbial interactions in higher attine symbioses, ultimately determining to what extent the interaction with *Escovopsis* would render detrimental for the colony (Figure 5).

It is uncertain which biological features make *Leucoagaricus* sp. MT more susceptible to *Escovopsis*. We hypothesize that susceptibility differences between *L. gongylophorus* AS and *Leucoagaricus* sp. MT could reflect the effectiveness of defensive mechanisms. Such approaches could involve fungal symbiont-secreted chitinases to avoid invasive microbes (Erthal et al., 2009), and laccases (Aylward et al., 2013; De Fine Licht et al., 2013) to detoxify *Escovopsis* metabolites with antimicrobial properties (Dean and Eriksson, 1994; Folgarait et al., 2011; Becker et al., 2016; Divya and Sadasivan, 2016; Boya et al., 2017; Dhodary et al., 2018; Heine et al., 2018). It is possible that the low laccase activity (De Fine Licht et al., 2013) would render *Leucoagaricus* sp. MT more susceptible to *Escovopsis* metabolites. Fungal cultivar laccases could also be involved in biosynthesis of heterogeneous melanin, known for protecting hyphae from toxins and hydrolytic enzymes (as chitinases and glucanases). Melanin accumulation results in a characteristic dark pigmentation with antimicrobial properties at interaction zones, which could be occurring in *in vitro* assays (Bull, 1970; Bell and Wheeler, 1986; Boddy and Hiscox, 2017; Figures 1C,D). Another potential defensive mechanism includes the biosynthesis of antimicrobial metabolites by the fungal cultivar (Hervey and Nair, 1979; Wang et al., 1999), which would partially explain the *Escovopsis* inhibition observed in 21% of *in vitro* interactions (Figure 1). However, these putative mechanisms seem not enough to outcompete *Escovopsis* growth, which was attracted to both fungal cultivars and inhibited their growth in the majority of FEI (Figure 1). It is worthy to point that fungal cultivar inhibition is not an outcome particular to interactions with *Escovopsis*, as it also results from *in vitro* interactions between cultivar and other filamentous fungi (Ortiz and Orduz, 2001; Silva et al., 2006; Barcoto et al., 2017; do Nascimento et al., 2017; Bizarria et al., 2018). Instead of being considered as a consequence of *Escovopsis* pathogenicity (Currie, 2001; Currie et al., 2003; Reynolds and Currie, 2004; Folgarait et al., 2011; Varanda-Haifig et al., 2017), we suggest that such outcomes also integrate the fungal cultivar responses. These fungal-fungal interactions would depend on to what extent the fungal cultivar and *Escovopsis* respond to and are affected by each other. Therefore, *Leucoagaricus* sp. MT would be less effective in inhibiting *Escovopsis* and more affected throughout the interaction.

Fungus gardens susceptibility may include several other players and factors influencing FEI dynamics. Thus, outcomes observed *in vitro* are not fully replicated, as only 5% of the subcolonies died when infected with *Escovopsis*. Although *Escovopsis* treatment damaged the garden at some extent, the majority of healthy subcolonies were able to gradually recover within 14 days (Figure 3). Such damage may result from both direct effects of *Escovopsis* growth and the workers’ effort to eliminate the high concentration of inoculated conidia, since fungus garden grooming and weeding increase when colonies face fungal infections (Currie, 2001; Currie and Stuart, 2001; Barcoto et al., 2017; Nilsson-Møller et al., 2018). As weeding implies the removal of fungus garden fragments, this defensive strategy could also reduce the garden lifetime. The initial damage followed by recovery could reflect multiple defensive barriers presented by a healthy colony, imposing limitations for the infectivity potential of *Escovopsis* (Figure 5). Defensive barriers to avoid accumulating damage would include: (i) ant workers cleaning and sanitizing behaviors (Currie and Stuart, 2001; Fernández-Marín et al., 2006); (ii) potential detoxification and antimicrobial mechanisms by the fungal cultivar (Hervey and Nair, 1979; Bell and Wheeler, 1986; Wang et al., 1999; Divya and Sadasivan, 2016; Boddy and Hiscox, 2017); and (iii) the garden microbiota (Santos et al., 2004; Egan and Gardiner, 2016; Longford et al., 2019; Barcoto et al., 2020).

When some of these defensive barriers are disrupted, for instance by insecticide treatment, the healthy functioning of the entire system appears to collapse (Figures 4, 5). Sulfluramid (N-ethyl perfluorooctane sulfonamide) is widely employed for eliminating leaf-cutting ant species in the field in Brazil. This insecticide acts by interrupting electron flow through the mitochondrial matrix, ultimately preventing aerobic respiration and causing the ants’ death (Boaretto and Forti, 1997; Della Lucia et al., 2014). With an increasing mortality of ants, multiple chemical and behavioral defenses would be simultaneously put down. For *Atta* ants, reducing the number of tending workers imply a decrease in glandular antimicrobial compounds (Fernández-Marín et al., 2006), as well as a lower frequency of fungus grooming and weeding for controlling and removing harmful microorganisms (Currie and Stuart, 2001; Barcoto et al., 2017; Nilsson-Møller et al., 2018). *Atta* species apparently lost cuticular Actinobacteria symbionts over the evolutionary time (Currie et al., 2006; Li et al., 2018), possibly relying on alternative behavioral and chemical defenses to control the growth of infectious microbes (Currie and Stuart, 2001; Fernández-Marín et al., 2009; Fernández-Marín et al., 2013; Yek et al., 2012). As a direct consequence of ant mortality, the absence of cleaning strategies would allow the spread of pathogens throughout the garden. In contrast, for the majority of other attine genera that present cuticular Actinobacteria, these symbionts may aid to the complexity of host-pathogen dynamics in the fungus garden (Sen et al., 2009; Goldstein and Klassen, 2020). These filamentous bacteria, mainly in the genera *Pseudonocardia* and *Amycolatopsis*, produce broad spectrum antimicrobial compounds thought to defend the workers and the fungus garden against diverse fungal antagonists, including *Escovopsis* (Currie et al., 1999b; Oh et al., 2009; Sen et al., 2009; Dângelo et al., 2016; Goldstein and Klassen, 2020). It would be very informative to evaluate host-*Escovopsis* dynamics throughout the attine group, comparing outcomes of interactions on Actinobacteria-hosting and non-hosting ant genera.

Ants mortality is accompanied by decrease in foraging activities (Figure 4), implying substantial reduction in plant substrate incorporation, which could affect the fungal cultivar metabolic activity (Schiøtt et al., 2010; Moller et al., 2011). Feeding a “vicious circle” (Beldomenico and Begon, 2010), this would impact the ants’ nutrition (Silva et al., 2003) and environmental conditions important for microbial community assembling (Barcoto et al., 2020). When reaching a threshold amount of disturbance, the fungus garden would be susceptible to virulence traits (i.e., features causing disease), exhibiting disease symptoms (Casadevall and Pirofski, 2001, 2003). Virulence traits would emerge from SEI only when the host health was previously disturbed (Casadevall and Pirofski, 2001, 2015; Brown et al., 2012; Méthot and Alizon, 2014). Under these circumstances, *Escovopsis* secondary compounds (Boya et al., 2017; Dhodary et al., 2018) and enzymes for metabolizing the mycelia of fungal cultivars (de Man et al., 2016), would enhance its infectivity and induce disease. Since the disease progressed only when the subcolony health was already impaired, *Escovopsis* infections seem to have an opportunistic nature (Casadevall and Pirofski, 1999; Brown et al., 2012; Méthot and Alizon, 2014; Casadevall and Pirofski, 2015). Rather than the effect of a single microbial agent, diseases in higher attine fungiculture could be a consequence of disturbances in the healthy microbial community (including the fungal cultivar; Casadevall and Pirofski, 2015; Egan and Gardiner, 2016; Biedermann and Rohlfs, 2017; Bass et al., 2019). Environmental disturbances would increase the fungus garden susceptibility, allowing the transition of commensals, saprotrophs, and opportunistic microbes to a pathogen mode (Méthot and Alizon, 2014; Egan and Gardiner, 2016; Bass et al., 2019). Therefore, when previously treated with sulfluramid, subcolonies were highly susceptible to *Escovopsis* infections, with health conditions impairing fast and progressively to death (Figures 4, 5). It is unclear to what extent and by which mechanisms *Escovopsis* negatively affected already debilitated subcolonies (Supplementary Table 7). Plausibly, other fungi reported in fungus gardens (as non-inoculated *Escovopsis*, *Escovopsioides*, *Syncephalastrum, Fusarium*, and *Trichoderma*, Supplementary Table 6) could have a similar effect in such conditions. Also, these fungi could add to the potential negative impact of *Escovopsis*, contributing to a cumulative detrimental situation.

*Escovopsis* has the capacity to metabolize diverse carbon sources (de Man et al., 2016). This would allow *Escovopsis* to function as a context-dependent commensal in healthy fungus gardens, potentially exploiting carbohydrates derived from the fungal cultivar metabolism (Gomes De Siqueira et al., 1998; Silva et al., 2003, Silva et al., 2006; Moller et al., 2011; Mathis and Bronstein, 2020). *Escovopsis* growth and infectivity would be limited by *Atta* ants’ cleaning and sanitizing behavior, as well as by the cultivar and the microbial community defenses. Environmental stresses or fungus-garden senescence would weaken the colonies defensive mechanisms, altering the interactions within the microbial community and ultimately enhancing host susceptibility (Casadevall and Pirofski, 2015; Egan and Gardiner, 2016; Casadevall, 2017; Bass et al., 2019). In such circumstances, *Escovopsis* growth would not be limited either by the ants, the fungal cultivar, or the microbial community. When the host becomes susceptible, the *Escovopsis* pathogenic transition would be triggered (Pitlik and Koren, 2017; Bass et al., 2019), enhancing its infectivity. *Escovopsis* pathogenicity would be based on degradation of the fungal cultivar mycelia (Reynolds and Currie, 2004) and production of metabolites toxic to the workers and the cultivar (de Man et al., 2016; Boya et al., 2017; Dhodary et al., 2018; Heine et al., 2018). For *Atta* fungus gardens, these effects could be correlated to decreasing in fungus garden biomass, amount of workers and brood reported as consequence of *Escovopsis* infections (Currie, 2001). Alternatively, as *Escovopsis* codifies for amylolytic and β-glucanolytic hydrolases, these enzymes would help in outcompeting the fungal symbiont for starch and glucans as carbon sources (Silva et al., 2006; Erthal et al., 2009; De Fine Licht and Boomsma, 2010; Moller et al., 2011), further inhibiting the fungal cultivar growth. In addition, older parts of the garden appear to stimulate *Escovopsis* conidia germination (Augustin et al., 2017), raising the possibility of conidia remaining dormant while the fungus garden is healthy, then germinating as the garden becomes older.

It remains to be evaluated the putative role of *Escovopsis* as a context-dependent commensal or saprotroph, as well as the metabolic processes behind the eventual pathogenic transition (Chamberlain et al., 2014; Bass et al., 2019; Mathis and Bronstein, 2020). Whether and which physiological processes could simultaneously sustain *Escovopsis* as a commensal and participate in the disease process (for instance, the chitinolytic and amylolytic capacity) are open research windows. It also remains to be investigated the resilience of *A. sexdens* symbiosis when interacting with *Escovopsis*, to define how much damage the colony tolerates before rendering a visible infection (Casadevall and Pirofski, 2015). Its worthy to consider that marked differences in caste and division of labor (Wilson, 1980), chemical and behavioral defensive strategies (Currie and Stuart, 2001; Fernández-Marín et al., 2006), and the apparent absence of cuticular Actinobacteria symbionts (Currie et al., 2006; Li et al., 2018) may render particular host-pathogen interactions in the *Atta* symbiosis. Whether and how patterns of host-modulated pathogenesis would emerge from complex interactions involving ant-Actinobacteria-fungal cultivar-*Escovopsis* in Actinobacteria-hosting attines is yet to be verified. A growing understanding of infectious diseases as outcomes of complex host-microbial interactions has evidenced that commensals and saprotrophs have the potential to turn into pathogens when having the opportunity to do so (Méthot and Alizon, 2014; Casadevall, 2017; Pitlik and Koren, 2017; Bass et al., 2019). For *A. sexdens* colonies-*Escovopsis* interactions, our findings suggest that the opportunity to become a disease-causing agent is modulated largely by the susceptibility of the fungal cultivar, the ants and the fungus garden.

## Data Availability Statement

The datasets presented in this study can be found in online repositories. The names of the repository/repositories and accession number(s) can be found in the article/Supplementary Material.

## Author Contributions

IJG designed the experimental setup, carried out the assays, the data analysis, drafted initial versions of the manuscript text and figures discussed the results, and contributed to the manuscript writting. MB designed the experimental setup, conceived the figures, discussed the results, and wrote the manuscript. QM carried out the assays and data analysis, discussed the results, and contributed to the manuscript writing. AG carried out the assays, discussed the results, and contributed to the manuscript writing. LM carried out the assays and data analysis. OB provided the ant colonies for the experiments, provided expertise in rearing ant colonies in the lab, discussed the results, and contributed to the manuscript writing. AR designed the experimental setup, assisted with results interpretation and discussion, and wrote the manuscript. All authors contributed to the article and approved the submitted version.

## Conflict of Interest

The authors declare that the research was conducted in the absence of any commercial or financial relationships that could be construed as a potential conflict of interest.
