# Supplementary material for: Host Susceptibility Modulates Escovopsis Pathogenic Potential in the Fungiculture of Higher Attine Ants
Source: Front Microbiol. 2021 Jun 14;12:673444. doi: 10.3389/fmicb.2021.673444 (PMC8238408; doi:10.3389/fmicb.2021.673444)

Supplementary Material

# Supplementary Data

Supplementary Tables S1-S8 are provided in an Excel file.

# Supplementary Figures

**
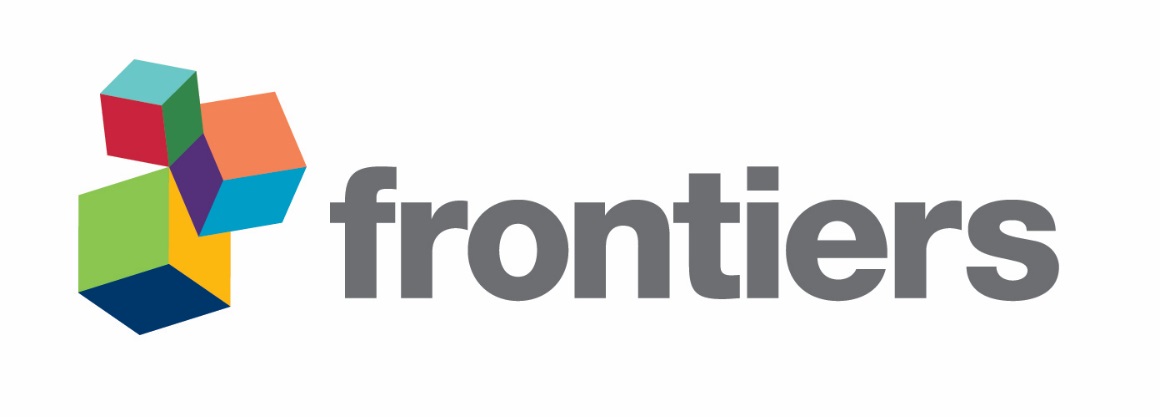
**

**Supplementary Figure 1.** Dual-culture *in vitro* assays for assessing the ant fungal cultivar-*Escovopsis* interactions (here exemplified by *Leucoagaricus gongylophorus-Escovopsis* confrontations). Control (*L. gongylophorus*): ant fungal cultivar after 14 days growing in PDA. Control (*Escovopsis*): *Escovopsis* after 7 days growing in PDA, inoculated in the beginning of the experiment (day 0). Co culture: Ant fungal cultivar (M) after growing for 14 days, and *Escovopsis* (E) inoculated in the beginning of the experiment (day 0).


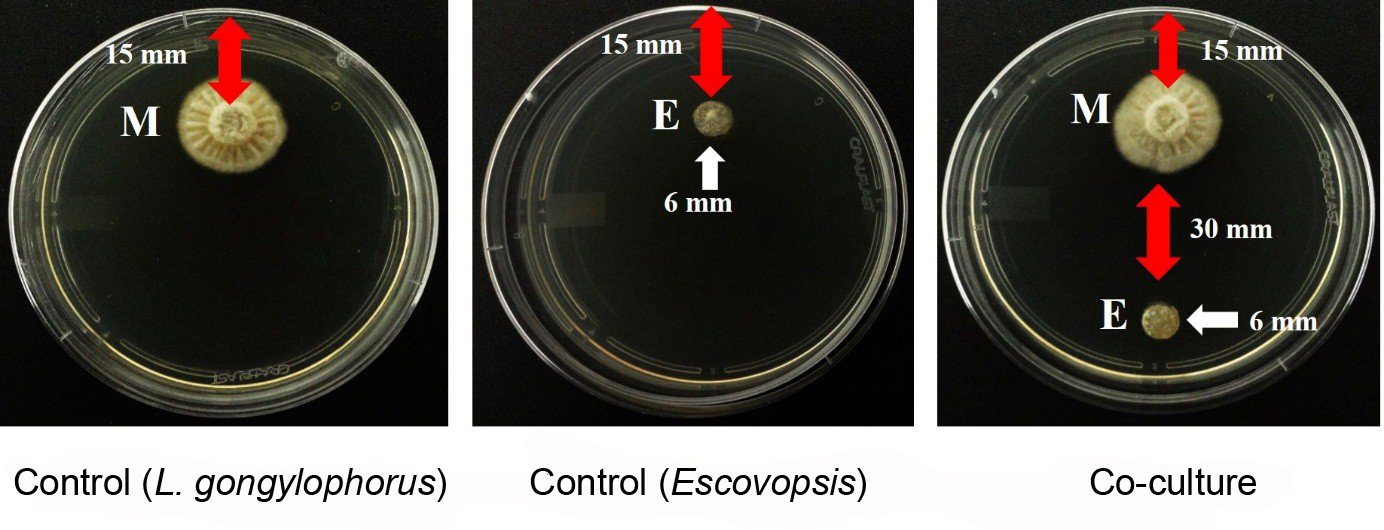


**Supplementary Figure 2.** Subcolony setup. Recipients (500 mL) for: A) Foraging; B) Fungus-garden chamber; C) Waste chamber.


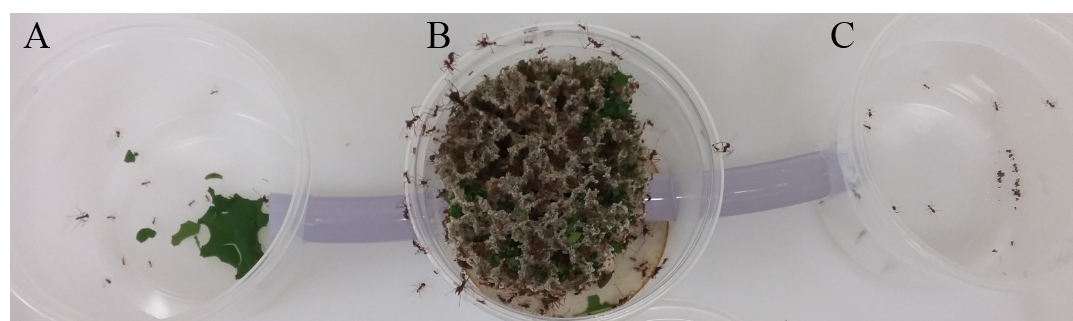


**Supplementary Figure 3.** Changes in the fungal cultivar morphology throughout confrontations, emphasizing mycelial loss of opacity. *Leucoagaricus* sp.-*Escovopsis* interactions are represented here.


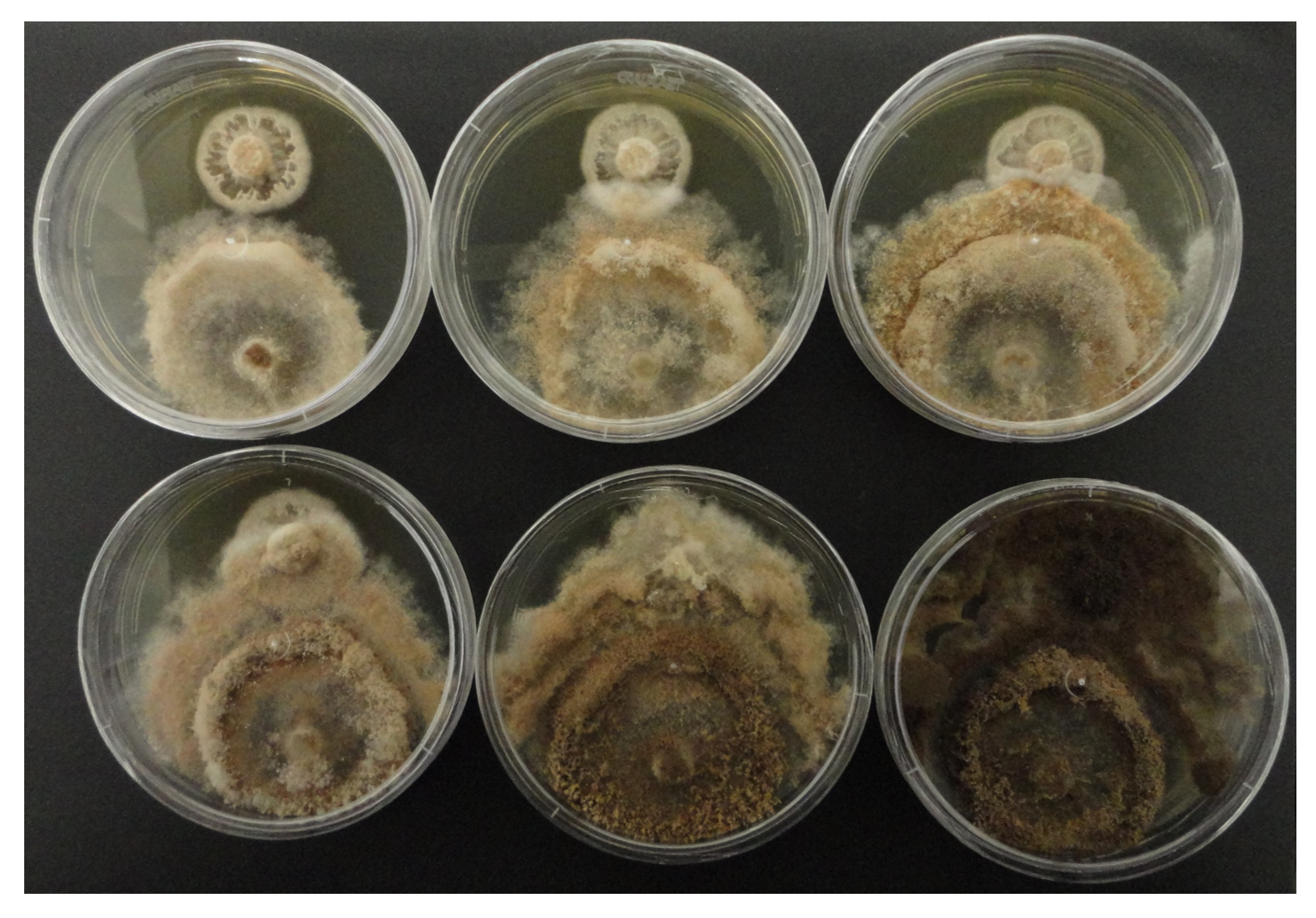

Supplement: Supplementary file 1 [file Table_1.DOCX]
